# Heatwaves, biodiversity and health in times of climate change

**DOI:** 10.1016/j.jped.2024.10.002

**Published:** 2024-10-30

**Authors:** Marcelo de Paula Corrêa

**Affiliations:** Instituto de Recursos Naturais, Universidade Federal de Itajubá, Itajubá, MG, Brazil

**Keywords:** Climate change, Public health, Social vulnerability, Infant mortality, Preventive policies

## Abstract

**Objectives:**

This article discusses heatwaves (HWs), their definitions, and increasing frequencies associated with climate change, as well as their effects on human health, especially on children and vulnerable groups. It emphasizes the need for interdisciplinary studies to better understand the effects of HWs and preventive actions to mitigate the effects caused by this phenomenon.

**Data source:**

The data were obtained from recent studies, conducted in Brazil and abroad, on the impacts of HWs. The figures were attained with data provided by the Climate Change Knowledge Portal.

**Data summary:**

HWs are periods of extreme heat, modulated by climate phenomena such as *El Niño* and the Pacific Decadal Oscillations. The frequency and intensity of HWs have increased since the 1950s, driven by climate change. HWs affect public health by increasing the risk of mortality from respiratory and cardiovascular diseases. Children are more vulnerable to problems such as fever caused by heatstroke, respiratory and kidney infections, as well as risks such as sudden infant death syndrome. Almost half of the HW episodes observed in South America in this century occurred in Brazil, mainly in socioeconomically vulnerable regions.

**Conclusions:**

The increase in the number of HWs is a direct consequence of climate change and has severe impacts on public health and biodiversity. Vulnerable groups suffer more from these phenomena, and social inequalities aggravate the problems. It is essential to promote awareness, implement effective public policies and encourage interdisciplinary research to mitigate the effects of HWs on society.

## Introduction

Heatwaves (HWs) can be defined as periods in which high temperatures accumulate over a sequence of days and nights considered exceptionally warm. There is no formal definition of the minimum time period or temperatures required to constitute a HW. For instance, the National Weather Service of the United States of America (USA) defines HW as a period of abnormally warm weather lasting at least two days or more, and with either high or low humidity.^1^ In Europe, EuroHEAT, a project coordinated by the Regional Office of the World Health Organization (WHO), provides a more technical definition: “HW is a period in which the apparent maximum and minimum temperatures are above the 90th percentile of the monthly distribution for at least two days”.^2^ There are also other related terminologies that can help indicate hot spells, such as the tropical nights index, defined as the annual number of days with a minimum nighttime temperature of at least 20 °C.^3^ In Brazil, the National Institute of Meteorology (INMET) adopts the recommendation of the World Meteorological Organization (WMO), which defines HW as the period of five or more consecutive days during which the maximum daily temperature exceeds the average maximum temperature by at least 5 °C.^4^^,^^5^

HWs are associated with increased risks in different sectors of society, mainly affecting the economy and health. Among the main effects are the increase in hospitalizations and human mortality, the duration and intensity of droughts, worsening water quality, forest fires and smoke, energy shortages, and agricultural losses, among others. The impact of HWs on mortality and morbidity affects all age groups. However, overall, children and the elderly are the most affected, in addition to people with specific medical conditions, particularly those suffering from cardiovascular and respiratory diseases.^6^ Recent studies carried out in Brazil show an association between the increased risk of mortality from different causes and the occurrence and intensity of HWs.^7^^,^^8^

The frequency and intensity of HWs have been increasing across the globe since the 1950s and there is scientific consensus that this trend is associated with climate change.^9^ These studies also converge on a finding of concern, indicating that the survival limit in several regions of the planet may be reached by the end of the 21st century due to the fatal combination of rising temperatures and extremes, positive or negative, of humidity.^10^ Human actions that are harmful to the environment, such as deforestation, contribute to the phenomenon of intensification in all regions of Brazil. For instance, the occurrence of more intense HWs in the Amazon in the last decade occurred during extreme droughts in the region and is associated with the reduction of the original forest area.^11^ This increase in HW episodes has also been recorded in recent decades throughout South America. In Brazil, in particular, a substantial contribution of persistent dry conditions to HW episodes has been observed, highlighting the vulnerability of the region to climate change.^12^

This article presents a brief overview of the most recent studies on the relationship between the occurrence of HWs, their relationship with biodiversity and their impacts on human health, especially on more vulnerable groups, such as children and adolescents. It is expected that this scientific review, although brief, will contribute to greater awareness on the subject, the development of preventive actions and the faster and more efficient identification to respond to medical emergencies that occur during such climatic episodes. Furthermore, the strengthening of knowledge about the dangers associated with HWs should always be seen as an incentive for the development of more interdisciplinary studies among researchers in the atmospheric and health sciences.

## Distribution, frequency and intensity of heat waves and their relationship with climate change

The year 2023 was the warmest year ever recorded in history.^13^ This claim is corroborated by meteorological records dating back to the 1850s and the reconstruction of a 2000-year time series using mathematical models.^14^ This record has also been confirmed by indirect evidence, such as studies of ice cores in Antarctica, indicating that it is possibly the warmest year in the last 100,000 years.^15^ Since its start, 2023 has also been a year of heat extremes occurring simultaneously in different parts of the world. For example, temperatures in some parts of Brazil exceeded 40 °C in mid-September, while much of Australia recorded temperatures 16 °C higher than normal ones. It is important to note that these high temperatures, and their early arrival in early spring, are consistent with projections made in previous studies. This increase in the occurrence of large-scale simultaneous extreme heat seasons is largely related to changes in atmospheric circulation due to global warming.^16^

HWs are expected to become more frequent, persistent and intense in almost all inhabited regions. These episodes are expected to accompany both soil drying trends, especially in mid-latitudes, as well as occurrences of humid HWs in other regions of the planet, such as in southern Asia, for example. These climate extremes also impact ecosystems and biodiversity through a greater predisposition to the occurrence of forest fires, commonly observed in Brazil, parts of South America, Australia, and the USA, among other countries.^17^

The consistency of these results is corroborated by studies that use a different range of analyses, databases and models, but whose results converge towards a future of changes in the severity, duration and frequency of HWs due to climate change. These projections indicate a strong impact on the most populous cities on the planet this century, as increases of between 3.4 and 6.6 °C in temperatures and HWs lasting between 4 and 10 days are expected. Even cities further away from the tropical and subtropical regions are expected to experience significant warming. For instance, Paris is expected to experience one of the most significant increases in the severity of HWs, with increases of 3.4 °C in HWs lasting 5 days and 1.7 °C in longer HWs lasting around 10 days.^18^

In South America, a study of 191 HW episodes observed in the region showed that approximately 47% of these hot spells were observed in the east and southeast of the continent; that is, within Brazilian territory. The hottest area, which extends from the northeast to the southwest of South America, stood out for its higher frequencies of intense HW episodes; and, across the continent, there was a significant increase in the intensity and persistence of HWs between 1979 and 2019.^19^ There is also significant evidence that HWs are becoming increasingly associated with droughts in northeastern and southeastern Brazil, in the Amazon and in the Pantanal.^20^ In a study carried out in the state of São Paulo, with observations of HWs occurring between 2000 and 2020, the average temperature of the HWs was approximately 35 °C, with an average duration of 5.3 days. It is important to note that 92% of these extreme events occurred between the springs and summers of the second decade of the studied period, that is, between 2010 and 2020.^21^

**Figure 1:**
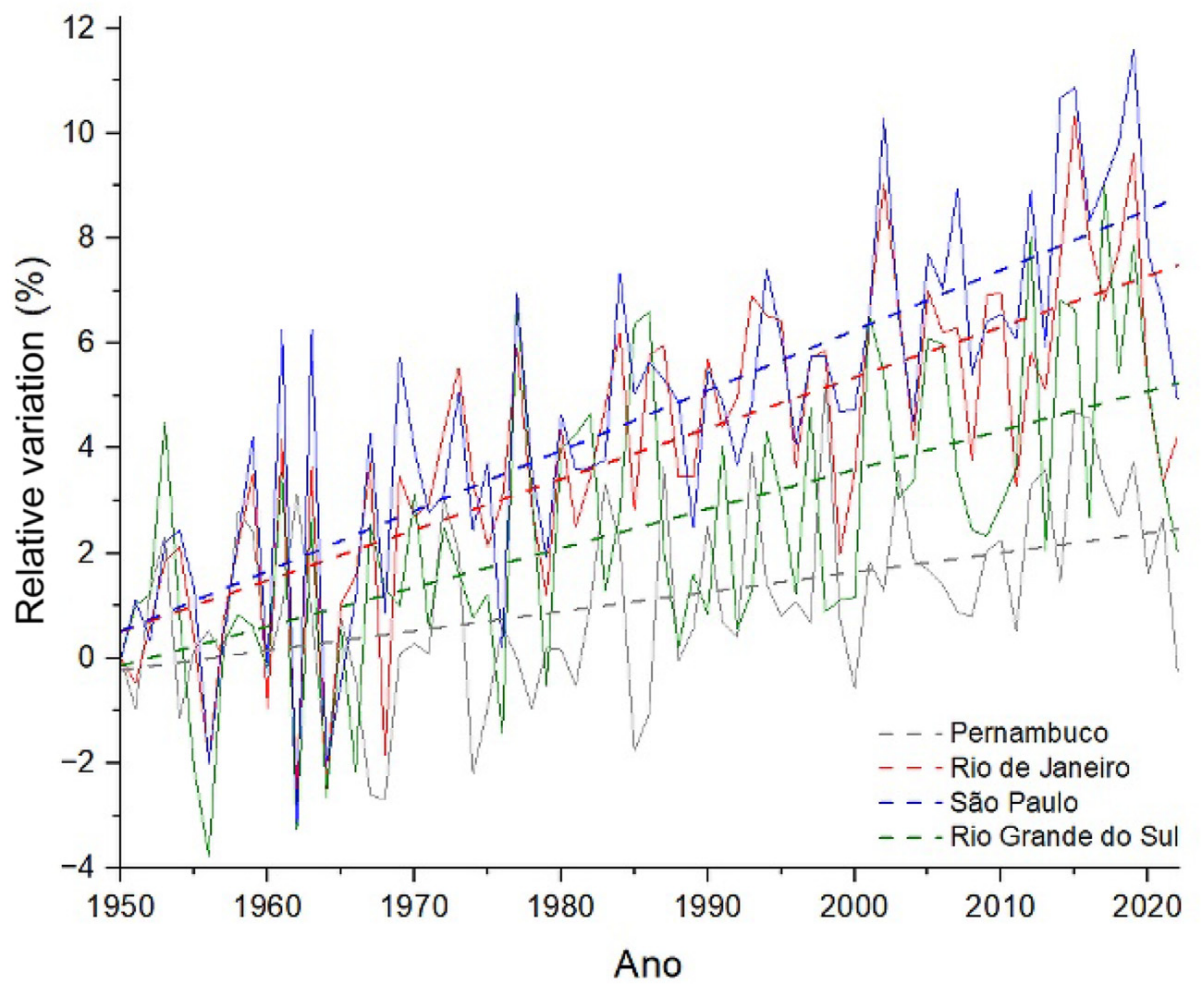
Relative variation (%) of the average maximum temperatures in relation to the year 1950 (solid lines). The dashed lines show the trend curve through linear regression of the data. Data source: World Bank.^22^

**Figure 2:**
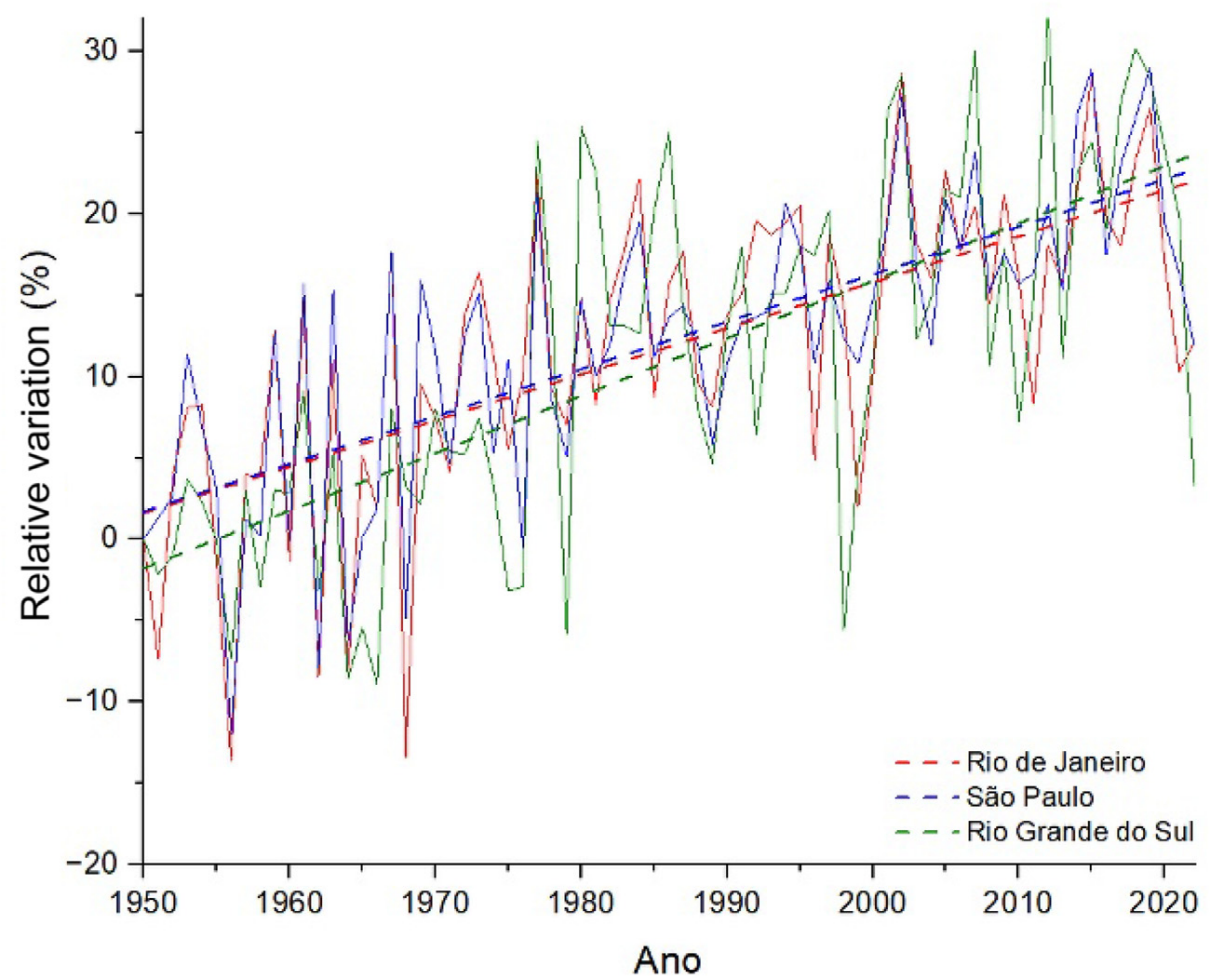
Relative variation (%) in the number of days in the year in which maximum temperatures exceeded 25 °C in relation to the year 1950 (solid lines). The dashed lines show the trend curve through linear regression of the data. Data source: World Bank.^22^

Figures 1 and 2 illustrate this trend of increasing temperatures, by showing the variation, in relation to the year 1950, of the average annual maximum temperatures ($\bar{T_{máx}}$) in four Brazilian states (Figure 1)^22^ and of the number of days in the year (N_T>25_) in the which maximum temperatures exceeded 25 °C (Figure 2).^22^ The relative differences (RD) depicted in the graphs were calculated as follows:

$$RD\;\left[\%\right]=100\;\times \left(\frac{x_{i}-x_{1950}}{x_{1950}}\right)$$

Where x represents and N_T>25_ in Figures 1 and 2, respectively.

Note that, in both figures, the linear regression curves of the time series (dashed lines) show a statistically significant increasing trend, (*p*-value < 0.001), both of and N_T>25_ between 1950 and 2020. That is, the average maximum temperatures increased between the mid-20th century and the beginning of this century in all regions of the country, with a greater increase in these temperatures in the southeast region of Brazil (blue and red curves). In parallel, the number of days with maximum temperatures above 25 °C increased between 20 and 30% in the south and southeast regions, therefore being a strong indication of the increase in the frequency and intensity of HWs in these regions. Figure 2 does not show data for the state of Pernambuco, as there was no significant increase in N_T>25_, since the maximum temperatures in the region were already above 25 °C for most of the year in 1950.

It is important to emphasize that the increases in the occurrence and intensity of HWs in Brazil are also closely related to cyclical phenomena involving positive or negative variations in the average temperatures of the Pacific Ocean. For example, the Pacific Decadal Oscillation (PDO) and El Niño-Southern Oscillation (ENSO) are capable of modulating HWs in different regions of Brazil and the world. The PDO and ENSO are similar phenomena but with distinct temporal variations. While the former has a climate variation lasting about one or two decades, ENSO usually lasts between six and 18 months.^23^^,^^24^ The southern region of Brazil generally has a greater number of HWs during the warm phase of the PDO (increase in surface temperatures of the Pacific Ocean), while HWs were more frequent in the southeast and midwest regions during the cold phase of the phenomenon and, therefore, related to the decrease in temperatures in the Pacific. Moreover, in the southern part of Brazil, the intensity and persistence of HWs did not change significantly between the two phases of the PDO, but the events have clearly been more intense and persistent in the cold phase.^25^

## Heat waves and children's and adolescents’ health

From pregnancy to old age, humans are exposed to the harmful effects of HWs. During pregnancy, these effects are mainly associated with the capacity for thermoregulation and physiological changes, with a consequent increase in the future burden of chronic diseases, both in mothers and their infants.^26^ Furthermore, the HWs and the record droughts recently observed in southeastern Brazil have resulted in an increase in fetal mortality and preterm births.^20^ Another study carried out in the country, involving a broad information database from more than 160 million inhabitants, indicated a positive association between the occurrence of HWs and the number of hospitalizations at any age. Regarding mortality, the evidence suggests a greater relationship between HWs and deaths caused by respiratory problems than by cardiovascular causes. Women and the elderly constitute the most vulnerable groups,^7^ whereas men seem to be more susceptible to dying from ischemic stroke during HW episodes.^8^

In parallel, the number of hospital admissions due to perinatal conditions was most strongly associated with HWs.^27^ There is scientific consensus that young children (< 5 years) and elderly individuals (> 65 years), as well as those with chronic or cardiopulmonary diseases, are more vulnerable to HWs, regardless of socioeconomic or geographic factors. Regarding the geographic factor, the tropical regions of the planet stand out, since the increase in temperature during the HWs, and due to climate change, has reached physiological tolerance limits and may become recurrent in the future.^28^ In addition to the risk of death, daily life under high temperatures reduces labor productivity, increases the risk of injuries and illnesses and, even if indirectly, is associated with an increase in the number of violent crimes, sexual assaults and homicides.^29^^,^^30^

These problems are further aggravated by the regional socioeconomic context, especially in a country marked by great social inequalities. In the first two decades of this century, approximately 50,000 deaths were attributed to the increasing number of HWs in Brazil, which represents more than 20 times the number of deaths related to landslides in the same period. This excess heat-related mortality tended to be greater in the poorest regions of the country, i.e., north, northeast and midwest regions, with a greater emphasis on people with lower educational levels, among blacks and brown people and, as already noted, among women and the elderly.^31^ There is an increasing number of recent publications reinforcing the inseparable nature of social vulnerability aspects in the assessment of the relationship between mortality in children under five years of age and climate change. After all, aggregating all-cause mortality results across multiple seasons and climate zones can be misleading about nuances in response to heat exposure.^32^

In the case of children and adolescents, excessive heat increases the risk of sudden infant death syndrome (SIDS), mental health problems, fluid and electrolyte imbalance, heatstroke fever, and the occurrence of infectious, respiratory, renal, and cardiac diseases.^33^^,^^34^ Among infants, more deaths are reported during periods of HWs, but conclusive evidence on the direct relationship between these periods of heat and infant mortality is still lacking.^35^ However, such mortality may also be indirectly affected by HWs, as studies show that deaths by drowning among the elderly^36^ and children between five and 14 years of age increase by more than 10% during episodes of prolonged heat.^37^ Table 1, adapted from Hicks et al. (2023),^38^ depicts a summary of the health risks associated with heat exposure.^39^^,^^40^

**Table 1:** Health risks associated with heat exposure (adapted from Hicks et al.^38^).

Issue	Problem	Severity	Symptoms	Signs	Prevention	Treatment
Thermoregulation^39^	Heat stress	Mild	Discomfort	Normal vital signs	Limit heat exposure Seek cool or air-conditioned shelter Ensure adequate hydration Avoid strenuous exercise Infants, young children, and adolescent athletes are at greatest risk and should be carefully monitored.	Cool, hydrated
	Heat exhaustion	Moderate	Thirst, headache, weakness, dizziness, syncope, vomiting, dehydration	Body temperature < 40 °C Tachycardia, hypotension There is no dysfunction of the central nervous system or end organs		Cooling down, resting
	Heat stroke	Serious, life-threatening	Neurological dysfunction, altered mental status, hematemesis, hematochezia, purpuric rash, and other symptoms of heat exhaustion	Body temperature > 40 °C Heart, liver and/or kidney failure, hypotension, arrhythmia, pupillary changes, tetany		Emergency medical care Immediate cold water immersion Continuous cooling Ventilatory support Manage end-organ damage
Loss of fluids and electrolytes	Dehydration	Variable	Thirst	Normal vital signs	Maintain adequate fluid intake	Adequate fluid and electrolyte intake
Burns	Sunburn	Variable	Erythema, heat, pain, swelling, blisters	Mainly superficial	Avoid sun exposure Use sunscreen	Prevention: shade, clothing coverage, use of sunscreen
	Thermal burn	Variable	Erythema, heat, pain, blisters, paleness, or no pain are warning signs of a full-thickness burn.	Superficial, partial or full-thickness burn	Check surfaces that may be hot before exposing children	Prevention: Check hot surfaces, including sand and pavement
Other issues	Asthma exacerbation	Variable	Shortness of breath, coughing, wheezing	Respiratory effort, hypoxemia	Avoid extreme heat, always have relief medication available	Asthma management^40^
	Heat rash	Mild	Erythematous skin rash (may be pruritic)	Heat rash, common in babies	Stay in a cool environment, avoid overdressing babies and children	Light and cool clothes
	Edema	Mild	Swelling of the hands and feet	Dependent edema	Stay in a cool environment	Cool and elevate affected areas
	Muscle cramps associated with exercise	Mild	“Heat cramps” can occur with high-intensity exercise in heat or cold environment They are more common with lack of physical conditioning, dehydration or poor acclimatization	Normal vital signs	Avoid strenuous or unusual exercise on hot days and in unfamiliar climates Ensure adequate hydration	Rest, hydration, cooling down and stretching

It is important to note that, among the several health risks depicted in Table 1, the potential risk of death associated with heatstroke stands out, in addition to implications that can result in insufficient vital organ function, such as the heart, liver and kidneys. Another highlight is the association of heat exposure with the worsening of skin rashes and common chronic diseases, such as asthma.

## Final considerations

HWs emerge as a growing climate phenomenon and a cause for concern, associated with climate change and environmental degradation. The increase in the frequency and intensity of HWs, as evidenced by historical data and future projections, has direct and severe consequences for public health and biodiversity. Vulnerable groups, such as children, the elderly, and people with pre-existing health conditions, are particularly affected, showing an increase in mortality and hospitalization rates. The intersection between the effects of HWs and socioeconomic factors further aggravates these problems, exposing the social inequalities that permeate the response to these extreme climate events.

In this scenario, raising awareness and the implementation of preventive measures are essential to attenuate the impacts of HWs. It is imperative that there be a joint effort between researchers from different disciplines, in addition to effective public policies that address both public health and environmental protection. Promoting interdisciplinary studies and adopting strategies to deal with the consequences of HWs are essential to ensure the safety and well-being of the populations, especially in vulnerable regions. Only through integrated actions will it be possible to face the challenges imposed by HWs and their effects on society. In common, most of the studies cited in this article reinforce the need for more in-depth research on the effects of climate change on health, especially pediatric health, including the establishment of new guidelines for protective factors that take into account subgroups of individuals who are more vulnerable.

## Author's contribution

Study design, data collection and writing of the manuscript.

## Conflicts of interest

The author declares no conflicts of interest.
